# Supplementary material for: Association of C-peptide and lipoprotein(a) as two predictors with cardiometabolic biomarkers in patients with type 2 diabetes in KERCADR population-based study
Source: PLoS One. 2022 May 24;17(5):e0268927. doi: 10.1371/journal.pone.0268927 (PMC9128999; doi:10.1371/journal.pone.0268927)
Supplement: Attachment — Submitted filename: Response to Reviewers.doc [file pone.0268927.s002.doc]

Dear Dr. Kanhaiya Singh

Academic Editor

PLOS ONE

Thank you so much for this occasion to answer peer review and revise our manuscript (Submission PONE-D-22-01443 **is revised).** The following, my written answer to honorable referees’ comments and fully manuscript revision was done. Thanks for helpful comments. All comments and suggestions were corrected and are in yellow highlighted.

**Reply to the respected academic editor and honorable** reviewers’ comments

| Reviewer Number | Original comments of the reviewer | Reply by the author(s) | Changes done on related page and section. |
| --- | --- | --- | --- |
| Academic editor | 1. Please ensure that your manuscript meets PLOS ONE's style requirements, including those for file naming. | Our manuscript (highlighted in yellow) was modified based on the PLoS ONE submission guideline. | Page title, Abstract, Tables, References, |
|  | 2. You indicated that you had ethical approval for your study. In your Methods section, please ensure you have also stated whether you obtained consent from parents or guardians of the minors included in the study or whether the research ethics committee or IRB specifically waived the need for their consent. | Thank you for your comment. The individuals aged between 15 to 75 years were enrolled in **the household survey by non-proportional to size one-stage cluster sampling** on Kerman province residences. The written informed consent was signed by all of the participants after ensuring their well understanding of the participation in the survey. Therefore, according to the age range of the patients, in our study, the patients themselves presented informed consent. | The underlined sentence added to first paragraph of “Materials and methods” |
|  | 3. We note that the grant information you provided in the ‘Funding Information’ and ‘Financial Disclosure’ sections do not match. | Thank you very much for your comment. As you know, The grant number of this research was (Grant ID: 95000643), however, the Ethics Certificate code was (IR.KMU.REC. 1395.938). | I will carefully revise. |
|  | 4a. Please include your amended statements within your cover letter; we will change the online submission form on your behalf.  b. We note that you have provided funding information that is currently declared in your Funding Statement. However, funding information should not appear in the Acknowledgments section or other areas of your manuscript. We will only publish funding information present in the Funding Statement section of the online submission form.  c. Please remove any funding-related text from the manuscript and let us know how you would like to update your Funding Statement. Currently, your Funding Statement reads as follows:   (NO. The Vice chancellor for Research of Kerman University of Medical Sciences had no role in study design, data collection and analysis, decision to publish, or preparation of the manuscript)  d. Please include your amended statements within your cover letter; we will change the online submission form on your behalf. | Thank you for your comment and description.  **a.** I modified the Acknowledgment in the text of manuscript and deleted the Funding source from it.  **b.** You are right. With permission, I deleted the Declaration from the text of manuscript and amended statements to Cover letter.  **c.** I removed any funding-related text from the manuscript.  I mean, the Vice chancellor for research had no role in how to buy the kits, study design, data collection, and make decisions in other cases (we hadn't any potential conflict of interest relevant to this manuscript).  **d.** All right. Thank you very much. | I modified it. |
|  | 5. In your Data Availability statement, you have not specified where the minimal data set underlying the results described in your manuscript can be found. PLOS defines a study's minimal data set as the underlying data used to reach the conclusions drawn in the manuscript and any additional data required to replicate the reported study findings in their entirety. All PLOS journals require that the minimal data set be made fully available. For more information about our data policy, please see http://journals.plos.org/plosone/s/data-availability. | I beg your pardon for any inconvenience. You are right.  I have a final SPSS dataset from analysis of our data. There is an Excel dataset file for Lipoprotein (A) and C-peptide data that had transferred from Dr. Yaser Masoumi-Ardakani to me.  I agree with your suggestion. I can transfer those files when resubmit our manuscript. | All data underlying the findings described in our manuscript fully available without restriction. These data include a SPSS dataset file and twelve related files from data analysis as well as an Excel file from lipoprotein(a) and c-peptide assay data. |
| Additional Editor Comments: | Although the reviewers have found this study interesting, they have raised some concern about the significance and novelty of this study. Please also elaborate about the guidelines being used to treat patients with T2DM in your population. | An updated and native version of the Diabetes Clinical Guideline consists of 150 pages in Persian has been published based on the general framework for providing services to diabetic patients in May 2021.  The guideline covers topics related to the diagnosis, prevention and care of diabetes, nutrition, education, insulin use, oral medication, control of dyslipidemia, treatment of hypertension, use of antiplatelet in diabetes, vaccination, treatment of hypoglycemia, prevention and treatment of diabetes complications, diagnosis and treatment of Gestational Diabetes, fasting, oral diseases, and Diabetes Referral Protocol for Psychological Issues.  Only one page is in English: A diagram about glucose-lowering medication in type 2 diabetes: 2021 ADA Professional Practice Committee (PPC) adaptation of Davies et al. |  |
| Major concerns of Reviewer 1 | 1. Giving clinical perspective to the paper: Existing medicines already are known to lower C-peptides and lipoprotein levels, so the study does little in terms of adding to the solution for these Laboratory test. Kindly mention what are the current guidelines followed in Middle East to treat T2DM | Thank you for your comments. I described to respected academic editor about an updated and native version of the Diabetes Clinical Guideline consists of 150 pages in Persian that compiled based on Evidence-based Medicine in Diabetes mellitus and perspectives and authorities of endocrinologist/internist.  Unfortunately, this guideline hasn’t any bibliographic identification in Iran’s National Library. However, I can’t refer to it in Reference section of manuscript.  Only one page is in English: A diagram about glucose-lowering medication in type 2 diabetes: 2021 ADA Professional Practice Committee (PPC) adaptation of Davies et al.  As you know, lipid and lipoprotein-lowering agents such as Ezetimibe, Fibrates, and Statins can influence on lowering lipoprotein (a).  I provided two new references about effectiveness medications on plasma Lp(a) levels (refs: 28 & 29 highlighted in yellow). These two new references and previous reference (27) and the other references could provide a clinical perspective.  With permission, I provided the authors’ perspective that compiled the updated and native version of diabetes clinical guideline in Persian about adherence of diabetic patients to their medications (ref. 30). | I added four references in Discussion section. Highlighted in yellow.  Lines 369-381. |
|  | 2. Methodology: Addressing the Latent period of pre-diabetes: consider giving latest references of how this is affecting the biomarker levels | Dear referee, as you know, one of the main aim was to find out whether there was an association between serum C-peptide and Lp(a) levels as two predictors with biomarkers of cardiometabolic disease risk in patients with T2DM and **healthy people** based on genders in KERCADR study as an Iranian community.  Let us not discuss these biomarkers in patients with prediabetes, due to the complexity of the topics. |  |
|  | 3. Novelty concerns: existing biomarker for management of Type 2 DM that is C-peptide level is well controlled with injectable Insulin and SGLT2 inhibitors are a class of prescription medicines that are FDA-approved for use with diet and exercise to lower blood sugar in adults with type 2 diabetes. Medicines in the SGLT2 inhibitor class include canagliflozin, dapagliflozin, and empagliflozin. Please mention these in the manuscript | You are Right. These medications were pointed out in the updated and native version of Diabetes Clinical Guideline. Clinicians occasionally prescribe these medications. Not everyone is able to buy these medications due to inflation and sanctions. |  |
| Minor concerns of Reviewer 1 | 1. Abbreviations need to be defined at the start of abstract: line 28: Lp(A), Line 34: AIP, CRI | Thank you very much for your comment. I modified based on PLoS ONE guideline submission. | Modifications and changes in Abstract section were added. |
|  | 2. Fig 1,2: AIP, CRI, CRII legends do not represent the abbreviations properly | Based on PLoS ONE guideline submission, I modified in text of manuscript and added title and legends to Figs 1 & 2. | Modification was done. |
| Major concerns of Reviewer 2 | 1. The authors mention that the data is fully available without restriction, but also mention that restrictions apply due to licensing issues. Please clarify this point. | Thank you for your comment. The data of this research (SPSS file) is fully available without restriction. |  |
|  | 2. Line 101-102, please briefly explain the study design here. Interested readers can look up the citations, however, a brief explanation here can help the readers move along this manuscript. | I briefly described the protocol. Highlighted in yellow. | Lines 102-109. |
|  | 3. Line 115-116, for the T2DM eligibility criteria, the authors mention ‘6) participants with T2DM receive either therapeutic diet or therapeutic diet with a combination of oral anti glycemic drugs’. Was this only for selecting participants, or were the study participants given this diet? I did not see this data point being used anywhere in the remaining manuscript, hence, the question. | Thank you for your comment. As you know and mentioned, **this criterion has been the criterion for selecting patients with type 2 diabetes**. We did not give them any meals. Only after blood sampling, a snack was given to all study participants, as well as their travel expenses. | Line 122 without highlighted in yellow. |
|  | 4. Materials and methods, for ‘Determination of serum lipoprotein (a)’, ELISA has been explained in detail, whereas, for ‘Determination of serum C-peptide’, that is not the case. Given the order of occurrence, details should be mentioned for serum C-peptide and in the case of Lp(a), authors can reference the C-peptide assay. | Dear referee, I shifted the subheading of lipoprotein(a) to subheading of C-peptide. | Lines 154 & 165. |
|  | 5. Line 376-377, the limitations of the study are not defined adequately. Dropping out participants was due to their ineligibility for the study. How is that a limitation? Please define. | As you know, Each study is designed based on the characteristics of the same study. Our study has been a nested case-control study, and I have described in “Participants eligibility and study design” section in details.  The proportion between cases and controls became 1:1 due to the precise control of confounding variables in the study. Hence, the study groups did not vary regarding specified and impressive confounders. Therefore, a matched healthy control was carefully chosen for each case from among participants in the KERCADR cohort study.  Therefore, we had to exclude many diabetic patients because the study has been a nested case-control study. I included some criteria in the inclusion criteria (eg. lack of high blood pressure, BMI lower than 30, ….). Hence, many diabetic patients had high blood pressure or BMI>30 and excluded from the study. These criteria were confounding variables for our study. | Lines 110-113 in “Participants eligibility and study design” section.  Lines 406-408 in Limitations. |
| Minor Points | 6. Line 392-393, please combine both the sentences. Also, kindly have a final concluding statement for the manuscript. | Thank you very much. I combined two sentences.  With permission, final concluding statement was provided in framework of a suggestion. | In conclusion. Highlighted in yellow. |
|  | 7. Line 56, should be ‘and has been revealed to display…’. | I modified it. The passive voice sentence converted to the active voice sentence. | Line 92, introduction. |
|  | 8. Line 58, should be ‘as a biomarker for…’. | Thank you for your comment. I modified it. | Line 60-61, introduction. |
|  | 9. Line 90, please define ‘CHD’. | Based on guideline submission, I defined CHD. | Line 59, introduction. |
|  | 10. Line 289, ‘on the one hand’, does not go along with the sentence. Please modify accordingly. | Thank you for your comment. I modified accordingly. | Line 307, paragraph 2, discussion. |
|  | 11. Line 383-384, should be ‘had exposure to…’. | Thank you for your comment. I modified it. | Line 415, conclusion. |
